# Tardive dyskinesia in patients treated with atypical antipsychotics: case series and brief review of etiologic and treatment considerations

**DOI:** 10.7573/dic.212259

**Published:** 2014-04-09

**Authors:** Jungjin Kim, Eric MacMaster, Thomas L Schwartz

**Affiliations:** SUNY Upstate Medical University, Department of Psychiatry, Syracuse, New York, USA

**Keywords:** tardive dyskinesia (TD), antipsychotic agents, dopamine receptor antagonists

## Abstract

Tardive dyskinesia (TD) is a disfiguring side-effect of antipsychotic medications that is potentially irreversible in affected patients. Newer atypical antipsychotics are felt by many to have a lower risk of TD. As a result, many clinicians may have developed a false sense of security when prescribing these medications. We report five cases of patients taking atypical antipsychotics who developed TD, review the risk of TD, its potential etiologic mechanisms, and treatment options available. The goal of this paper is to alert the reader to continue to be diligent in obtaining informed consent and monitoring for the onset of TD in patients taking atypical antipsychotics.

## Introduction

Tardive dyskinesia (TD) is a delayed-onset involuntary movement disorder associated with antipsychotic medications. TD typically presents as orofacial dyskinesia but may also present as athetosis, dystonia, chorea, motor tics and less frequently myoclonus or tremor. TD does not present as parkinsonism-based resting tremor. While it commonly affects the lips, tongue and mouth, it also may involve other areas of the body. Reports of TD have been closely tied to the prolonged use of first-generation antipsychotics (FGA) ever since their introduction in the 1950s [1]. Although we still do not fully understand why TD occurs, antipsychotic-induced up-regulation of dopamine-2 (D2) receptors is one of the proposed etiologic mechanisms. However, this disfiguring side-effect has been noted to persist chronically, even after FGAs are withdrawn and receptors have down-regulated back to baseline [2].

In the 1990s, second-generation (atypical) antipsychotics (SGA) were introduced and quickly gained prescribing popularity, because they appeared to have lower risk of causing extrapyramidal symptoms (EPS) and TD as compared to the FGAs [3]. SGAs have since largely replaced the use of FGAs. However, SGAs have also been associated with the development of TD, and thus TD remains a real risk when using any antipsychotic therapy [4]. Furthermore, SGAs have come under clinical scrutiny and have evoked class action lawsuit situations due to their risk of inducing metabolic disorder (weight gain, hyperlipidemia, glucosemia and hypertension), thereby drawing clinicians’ attention to this set of side-effects and potentially away from monitoring for TD onset.

There is also a ‘third-generation’, or ‘atypical-atypical’ antipsychotic medication, called aripiprazole, which acts alternatively through either D2 receptor partial agonism or D2 functional selectivity [5]. Despite having one of the highest D2 receptor affinities at therapeutic doses amongst the SGAs, aripiprazole has a lower risk of causing acute extrapyramidal symptoms and TD, probably due to its partial D2 agonist properties [6–9]. Nevertheless, aripiprazole has also been reported to induce TD [5,10,11].

A typical clinical course of TD is marked by insidious onset, usually after 1–2 years of continuous exposure to antipsychotics. It subsequently develops into a full syndrome, followed by persistent symptomatology stabilization and typically can persist for years, even after discontinuation of the antipsychotic drug [12]. Spontaneous remission of TD 2 years after discontinuation of antipsychotics has been reported to be 33% [13]. This also means that TD may continue to some degree in a sizeable minority of patients at any level from mild to severe symptom severity.

Unfortunately, TD may be irreversible in up to 50% of affected patients [14] and has been shown in schizophrenics to lead to lower quality of life, treatment-refractory courses of illness and greater mortality [15,16]. Given the increased use of SGAs in today’s clinical practice, including frequent off-label use for non-psychotic conditions (major depressive disorder, personality disorder, autism spectrum disorder, sleep disorder, etc.), the occurrence of TD and its burden may continue to rise. This review utilizes a case series to alert the reader to the risk of TD development in a variety of patients who were treated with SGAs.

## Case series

### Case 1

A 46-year-old woman first presented to the outpatient clinic with a longstanding history of recurrent major depressive disorder (MDD). She reported symptoms of increased sleep, low mood, decreased interest, poor concentration, guilt and occasional passive suicidal ideation. She also admitted to visual hallucinations. She denied symptoms of mania, anxiety, or substance abuse. At initial presentation, she had been taking duloxetine, a selective serotonin-norepinephrine reuptake inhibitor (SNRI) antidepressant, at 120 mg per day for the past 2 years. Duloxetine had only slightly improved her mood.

She was initially prescribed off-label ziprasidone augmentation up to 80 mg per day. One month later, ziprasidone was discontinued due to akathisia. She was then prescribed aripiprazole (5 mg per day) along with duloxetine. She gained near-total remission of her psychotic MDD symptoms for the next 4 months. However, she then presented with involuntary lateral jaw movements. Aripiprazole was discontinued and she was started on buspirone (15 mg per day) augmentation to sustain depressive remission. Over the following 4 months, her TD symptoms gradually improved and remitted, but her depression returned. The medication regimen was then switched to desvenlafexine (200 mg per day) monotherapy. The patient currently reports improved mood and has not had recurrence of dyskinesias for 4 years.

### Case 2

A 55-year-old man with bipolar mania had a 16-year history of MDD episodes prior to his first visit. These were initially controlled with paroxetine (20–40 mg per day), a selective serotonin reuptake inhibitor (SSRI) antidepressant, but he eventually became resistant. He had failed multiple other antidepressants thereafter as they induced hypomanic states. He had also failed therapeutic trials of quetiapine, risperidone and olanzapine due to sedation and weight gain. The patient was placed on lamotrigine at his initial clinic visit and had good control of depressive symptoms for the next 7 weeks. At the end of the seventh week, however, he became manic.

He was next titrated onto ziprasidone (160 mg per day) as monotherapy. Despite this, he developed worsened mania over the next week and was hospitalized. While an inpatient, his ziprasidone dose was doubled to the off-label dose of 320 mg per day. His mania remitted after 2 weeks, and he was discharged. A few weeks after discharge, however, he noticed bilateral jaw twitching, along with lip puckering and smacking. Benztropine was started by another provider and did not relieve, nor worsen his abnormal movements. Suspecting TD, he was gradually tapered off ziprasidone. Approximately 4–5 months after stopping ziprasidone, his TD symptoms had largely improved to where he evidenced only minor and infrequent lip pursing movements. He has since been placed on carbamazepine (800 mg per day) with good tolerance and without any manic episodes for 2 years.

### Case 3

An 82-year-old woman with a history of lifelong MDD, characterized by depression-dependent anxiety and fleeting, passive suicidal ideation, presented to our clinic with 18 months of active depression without remission. She denied symptoms of psychosis, mania, or dementia. She had failed therapeutic trials of paroxetine, bupropion, venlafaxine and duloxetine in the recent past.

She was initially maintained on a combination of clonazepam (3 mg per day) and mirtazapine (30 mg per day) but she was still partially symptomatic after 4 months of treatment. In addition to this combination, she was given an SGA, aripiprazole, up to 15 mg per day. After 4–5 months, however, she developed blepharospasm and facial grimacing. Given her TD, she was tapered off aripiprazole over the next 8 weeks while the clonazepam/mirtazapine combination therapy continued. Dyskinetic movements remitted over the next 5–6 months.

Six months after stopping aripiprazole, however, she relapsed into severe MDD. She was started on the SGA quetiapine (up to 600 mg per day), but after 7 months, she redeveloped blepharospasm and facial grimacing. She was tapered off quetiapine as well, and her TD remitted. Since that time, she has received trazodone, lorazepam and electroconvulsive therapy (ECT) therapy with good control of her MDD symptoms while her TD has continued to be in remission.

### Case 4

A 67-year-old man with longstanding cyclothymic disorder and generalized anxiety disorder presented with symptoms of lip wetting for 1 year. For the past 7 years, he had been taking aripiprazole (10 mg per day) and tiagabine (4–24 mg per day) with a very good response, but not full remission of anxiety. Buspirone (15 mg per day) was added 3 years later to better this response. His regimen was continued with good symptomatic control (remission) for the next 4 years. He was still on these medications until his initial presentation.

When describing his lip wetting, the patient attributed it to chronic chapped lips which were relieved by using over-the-counter remedies. Suspecting that these movements were TD, our clinic tapered off his aripiprazole and left him on the tiagabine/buspirone combination therapy. Three months after stopping aripiprazole, his anxiety and mood symptoms remained under control. However, he continued to have lip smacking and lip wetting. He also developed tongue protrusions during this period. Given that his TD worsened after stopping aripiprazole, we now suspected withdrawal TD. To treat TD, we attempted trials of firstly branched-chain amino acid (BCAA) therapy consisting of leucine, isoleucine and valine three times daily (222 mg per kg per day; in a ratio of 4:3:3); secondly ginkgo biloba (240 mg per day); and finally vitamin E (1600 IU) for 2 months. None of these trials remarkably improved his TD and they were subsequently discontinued.

Nevertheless, his TD symptoms gradually improved over the next 6–12 months to the point of experiencing only occasional lip movements at rest. Interestingly, none of his TD movements occurred on abnormal involuntary movement scale (AIMS) testing, nor during activating tasks.

### Case 5

A 71-year-old female with a history significant for bipolar II disorder and obsessive-compulsive disorder (OCD), well controlled on quetiapine (600 mg per day) and fluoxetine (80 mg per day) for the past 10 years, presented to us with complaints of lip pursing and licking ascribed to her new dental bridgework. Suspecting TD, however, we lowered her quetiapine by half. Over the next 3 months, her perioral movements partially improved.

She would have at most 1–2 abnormal lip movements during her 30-minute visits and only upon activation tasks on AIMS testing. Her OCD symptoms were under good control with increased cognitive behavioral therapy (CBT) sessions despite the lowered quetiapine dose of 200 mg per day.

For the next 3 months, we further lowered her quetiapine to 50 mg per day but she continued her lip movements and additionally developed lateral tongue movements. In light of worsened TD despite lowered quetiapine, we suspected withdrawal TD and stopped quetiapine. Her mood was slightly elevated but was not hypomanic. Due to increased risk of hypomania, we lowered her fluoxetine (SSRI) dose by half to 40 mg per day.

Three months after stopping quetiapine, her TD symptoms had largely remitted. Her mood was slightly depressed, however, so fluoxetine was increased back to 80 mg per day and trazodone (50–100 mg per day) was added to the regimen. Lamotrigine (200 mg per day) was also added for maintenance protection against hypomania. She has since been euthymic with good control of anxiety for 12 months. No abnormal movements have been noted several months after stopping the antipsychotic agent.

## Discussion

### Incidence and prevalence of TD

TD prevalence and incidence rates are considerably variable depending on patient population, study design, duration and types of antipsychotic exposures. However, the largest literature review to date involved 34,555 patients treated with antipsychotics across 56 studies showing an average TD prevalence of 20% [17]. This study did not directly compare the prevalence rate for FGAs and SGAs, but the 2008 update to this study involved 2088 patients and found the prevalence rates of TD to be 13.1% with SGAs and 32.4% with FGAs [4].

TD incidence rate, on the other hand, is more difficult to obtain because it requires continuous monitoring of patients over time. Nevertheless, a meta-analysis of 12 studies from 2008 reported that the annual TD incidence rate is 2.98% with SGAs versus 7.7% with FGAs across 12 trials involving 28,051 patients [4]. The data suggest that the risk of TD has not been eliminated with SGAs, but is at least lowered.

These rates may yet be an underestimation of the true risk of TD. Medication non-compliance in schizophrenic patients has been reported to be as high as 50% [18], suggesting that patients may develop TD with much less exposure to antipsychotics. Furthermore, antipsychotics are known to induce bradykinesia and rigidity, which may mask the clinical presentation of TD by increasing muscle stiffness and rigidity, thus lowering the choreoathetotic movements associated with TD.

Multiple risk factors for TD have been identified, including old age [19], female gender [20], African-American race [21] and presence of affective disorder [22]. This latter variable was present in all of the five cases presented above. More often, the SGAs are being used in greater quantities in affective disorder patients and for longer durations to help maintain remission and affective stability. Of note, these risk findings are largely based on time-limited studies of TD prevalence and old age has been most consistently noted to be the strongest risk factor for TD. Of note, our patients were all middle aged to geriatric in age, suggesting that most of our patients carried major risk factors. Both higher incidence and lower remission rates are observed in patients older than 50 years of age [19]. As TD risk remains high in older adults who have affective disorders, a very high level of caution is suggested and warranted when prescribing antipsychotics in this population.

### Pathophysiology of TD

We still do not fully understand the pathophysiology of TD. Several theories have been proposed: (1) dopamine-2 (D2) receptor hypersensitivity; (2) oxidative stress created from chronic antipsychotic use; (3) dysfunctional striatal GABA input to motor neurons; (4) lower expression of serotonin (5HT-2A) receptors; (5) overall genetic susceptibility; and (6) lack of antipsychotic-metabolizing enzymes.

The most popular theory is the D2 receptor hypersensitivity theory [23–26]. D2 receptors are expressed on the inhibitory medium spiny neurons (MSN) that feed into the indirect dopamine pathway in the basal ganglia. Antipsychotics, via D2 blockade, are thought to lift the inhibition of dopaminergic neuronal output from both the globus pallidus and substantia nigra, which in turn leads to hyperkinetic movements [27]. Teo et al. have recently proposed a maladaptive synaptic plasticity hypothesis [27], which suggests that chronic antipsychotic use leads to D2 receptor hypersensitization on MSN interneurons, potentiating secondary changes in the synaptic plasticity of glutamatergic synapses on striatal interneurons. This produces an imbalance of direct and indirect dopamine pathway output to the sensorimotor cortex. This, together with abnormal basal ganglia output, may lead to miscoded motor programs and abnormal movements.

Long-term antipsychotic use, in particular, is thought to cause chronic antagonism, or blockade, of D2 receptors with gradual hypersensitization and up-regulation of the remaining D2 receptors [25]. Notably, FGAs have been shown to remain attached to the D2 receptors much longer than SGAs [28], and this may explain the higher rate of TD with FGAs.

In support of this theory, higher doses of haloperidol administration have been shown to temporarily suppress TD, whereas abrupt discontinuation of the agent has been shown to exacerbate TD [29,30]. Furthermore, D2 receptor density has been shown to increase after 2 weeks of haloperidol administration in rats [31] and increased chewing movements were associated with high D2 receptor occupancy as well [32]. Evidence in humans is not as robust but chronic antipsychotic use has been suggested to facilitate enhanced striatal D2 receptor binding and possibly TD. A recent DAT (dopamine transporter) scan with SPECT imaging in a schizophrenic TD patient further delineated that increased dopamine transporter uptake activity can be linked to TD improvement [33,34].

Another popular theory is the notion of striatal neurodegeneration from oxidative stress. Chronic antipsychotic dosing is thought to facilitate increased dopamine turnover and this, in turn, leads to production of high levels of free radicals [35–37]. The resultant oxidative environment is toxic to striatal neurons, causing neuronal loss and gliosis within the basal ganglia. This may explain cases of TD that persist long after antipsychotic discontinuation. Supporting this theory are reports of increased lipid peroxidation in the cerebrospinal fluid of TD patients [38] and modest anti-TD benefit demonstrated by antioxidants, such as vitamin E, in certain studies [39–41]. Furthermore, elevated levels of manganese superoxide dismutase, a key antioxidant enzyme, have been reported in TD patients and were correlated with TD severity [42].

However, systematic reviews of small-scale trials involving antioxidants, including vitamin E, have not clearly demonstrated anti-TD benefit [41]. Furthermore, neuroimaging studies of basal ganglia volumes of patients on antipsychotics were inconclusive; there was little difference in the basal ganglia volume of patients with and without TD [43]. Direct pathologic evidence of neurodegeneration from animal or human TD models is lacking as well.

Dysfunctional striatal GABAergic interneurons also have been suggested as the basis of TD symptom formation [44–47]. GABAergic interneurons provide key inhibitory input at MSNs [45] and these neurons fine-tune the inhibitory command necessary for focused movement execution [46]. Selective inhibition of these neurons within rat striatum has been shown to induce TD-like movements [47]. Long-term antipsychotic use and resulting D2 blockade, in theory, does damage to these GABAergic interneurons via glutamate-mediated excitotoxicity and increased oxidative stress from dopamine turnover [48]. This theory has gained support from observations that long-term antipsychotic treatment of primates has been associated with both dyskinetic movements and decreased basal ganglia GABA and GABA-synthesizing enzyme levels [49].

Another hypothesis is that of decreased availability of serotonin (5HT-2A) receptors. 5HT-2A receptors are widely distributed in the caudate and putamen of the dorsal striatum [50] and via complex interplay with D2 receptors, contribute to the regulation of motor activity [51]. 5HT-2A receptor gene (HTR2A) polymorphisms have been associated with increased risk for TD (odds ratio [OR]=1.6) across six studies and was specifically associated with limb-truncal TD [52].

In addition to the 5HT-2A receptor gene, a number of other candidate genes have been studied. Polymorphisms in a dopamine D2 receptor gene [53], a D3 receptor (DRD3) gene [54], manganese superoxide dismutase gene [42] and COMT genes [55] have been associated with varying degrees of influence on TD susceptibility. Though still not well understood, a complex interplay of genes appears likely to be involved in TD pathogenesis.

Lastly, the inability to metabolize antipsychotics has been associated with TD. Cytochrome P450 enzymes are known to facilitate metabolism of antipsychotics. CYP450 2D6 [56] and 1A2 [57] have been associated with a decreased ability to metabolize antipsychotics, theoretically leading to excessive plasma levels, receptor binding and drug accumulation of antipsychotics with dose-dependent elevation of TD risk. In support of this theory, one meta-analysis study showed that the loss of a functional CYP2D6 allele increased the risk of TD (OR=1.43) and the loss of both copies in homozygous individuals – poor metabolizers – carried a 1.64-fold greater risk of developing TD [58]. Conflicting reports, however, have been published since [59]. Overall, the genetic basis of TD, including CYP enzyme systems, remains incomplete, but is nonetheless an active area of ongoing investigation.

### Treatment of TD

Numerous pharmacologic agents have been investigated for the treatment of TD. A very good comprehensive review of evidence-based recommendations for the management of tardive syndromes, including TD, has recently been published by the American Academy of Neurology (AAN) [60] and we will defer detailed, systematic reviews of each of the management options to this literature. We will, however, highlight a few of these antidyskinesia agents in an effort to quickly educate the reader regarding the available options for treating TD.

Vitamin E is an antioxidant and is thought to counteract the free radicals created from chronic antipsychotic use. Small-scale trials have reported variable anti-TD benefit with vitamin E [39,40]. In 2001, however, a systematic review involving 256 patients reported no clinical benefit of using vitamin E [41].

Data on ginkgo biloba is limited, but a recent randomized controlled trial (RCT) of ginkgo biloba extract (EGb-761) on 157 Chinese schizophrenic patients showed marked improvement of TD symptoms (51.3% in treated versus 5.1% untreated) [61]. However, it should be noted that most patients were taking clozapine or were reported to have developed TD while actually taking this medication. Whether this result could be extended to TD induced by other antipsychotics remains unknown. Nevertheless, due to the above findings, the AAN suggests that a trial of EGb-761 may be helpful for the treatment of TD (class B recommendation) [60].

Previous studies have also examined amantadine, a non-competitive NMDA receptor blocker, because NMDA receptor-mediated excitotoxicity is one of the proposed pathomechanisms of TD. Amantadine has been suggested to dampen the excitatory glutamatergic signalling from the subthalamic nucleus to the internal globus pallidus [62], which could explain its antidyskinetic properties as demonstrated in multiple controlled and uncontrolled studies [62–66]. In one RCT, amantadine reduced TD during the first 7 weeks when used conjointly with neuroleptics [63]. Amantadine, together with neuroleptics, has thus been recommended by the AAN for the short-term treatment of TD [60].

Although reduced GABA levels can cause dyskinesias in primates [44], various GABA agonists – baclofen, gamma-vinyl-GABA, gamma-acetylenic-GABA, muscimol, tetrahydroisoxazolopyridinol (THIP) and sodium valproate – have been shown to offer little clinical benefit [67, 68]. Benzodiazepines, such as clonazepam, have also been tried due to their GABA agonist properties [69]. In an RCT involving 19 patients, clonazepam showed significant reduction of dyskinesia and dystonia [70]. Its antidyskinetic effects, however, disappeared after 5–8 months of continuous treatment. Based on this study, clonazepam has been recommended by the AAN for short-term (approximately 3 months) treatment of TD (class B recommendation) [60]. Lastly, a case report of TD improvement with the non-benzodiazepine GABA receptor agonist zolpidem has been published as well [71]. Due to limited data and potential for addiction, however, benzodiazepines should be used with caution at the discretion of the clinician as there are often comorbid addiction and psychomotor impairment issues noted in patients suffering from TD.

Modulation of acetylcholine and dopamine levels has been tried as well. Cholinergic agents including choline, lecithin, physostigmine, tacrine, rivastigmine, deanol, meclofenoxate and galantamine have been tried with limited success [72]. Galantamine, in particular, was associated with increased parkinsonism [73]. Donepezil is the only cholinergic agent with reports of some clinical efficacy against TD in preliminary open-label trials [74,75]. Evidence for the use of anticholinergics, such as benztropine, biperiden, chlorprothixene and trihexyphenidyl is not only limited, but has also been associated with the exacerbation of TD [76]. Physicians all too commonly equate TD and parkinsonism, treating TD with anticholinergics, but this may lead to exacerbation of TD (while alleviating parkinsonism).

Dopamine agonists (such as bromocriptine) and dopamine breakdown inhibitors (such as selegiline) were shown to have little clinical benefit [77,78]. Among dopamine depleting agents, tetrabenazine consistently reduced TD across two RCT studies, one comparing haloperidol use with tetrabenazine use (100 mg per day for 14 days) [79], and the other a non-randomized, single-blind study [80], and tetrabenazine was particularly effective for tardive dystonia [79–82]. Tetrabenazine is recommended by the AAN for the treatment of TD (level C recommendation) [60]. Reports of efficacy for reserpine and alpha-methyl dopa are available from a small RCT as well [83] but these drugs are not readily available and are associated with numerous adverse effects and typically are not utilized. Of note, buspirone, a serotonin receptor agonist, has been associated with improved TD in one study and was suggested to act via dopaminergic effects secondary to 5HT-1A receptor agonism [84].

Levetiracetam has been shown to be a promising agent in both of its open-label trials [85,86] and a case report [87]. One RCT reported significant TD improvement with 12-week treatment of dose up to 3000 mg per day, but the dropout rate was greater than 20% [88]. Overall there may be insufficient data supporting the use of levetiracetam for TD and this domain remains an active area of clinical investigation.

Branched-chain amino acids (BCAAs) have been shown to improve TD in RCTs in adult males [89] and in a small number of children and adolescents [90]. The improvement of TD with BCAAs was associated with reduced levels of phenylalanine, a dopamine precursor, in the brain [91]. This observation was used to suggest that BCAAs may be interfering with monoamine neurotransmitter synthesis, thereby lowering catecholamine and indolamine levels that may drive TD pathogenesis [89–91].

Use of botulinum toxin injections has largely been limited to localized forms of TD, particularly with dystonic features such as retrocollis, or blepharospasm [92]. Evidence supporting the use of botulinum toxin, however, is currently limited to open-label retrospective case series and case reports [60].

Lastly, surgical interventions remain an option for TD refractory to pharmacologic treatment. Deep brain stimulation of globus pallidus interna and lesioning surgeries like pallidotomy have been associated with various levels of clinical TD improvements [93–96]. The AAN has concluded that these data are insufficient to support or refute these surgical modalities [60]. In general, however, surgical treatments are reserved for severe, treatment-refractory cases of TD [97].

Despite these efforts, no clear or definitive treatment for TD exists to this date. Informed consent at antipsychotic treatment initiation, early detection of TD, and rational removal of the FGA or SGA while waiting for D2 receptor normalization and restoration of the nigrostriatal dopamine pathway functioning is the standard of treatment. Some symptomatic treatments may be tried as outlined above as a secondary option. In particular, clonazepam and ginkgo biloba probably should be tried initially (both level B recommendations), followed by amantadine and tetrabenazine (level C recommendation) [60]. Importantly, clinicians may consider referring refractory cases to a movement disorders specialist.

Nevertheless, providing good patient education at the start of treatment, monitoring for initial TD onset and early intervention remains the centerpiece of TD management. Clinicians may consider alternate augmentation and polypharmacy strategies when dealing with more elderly affective disorder patients as these patients seem to carry the greatest risk of TD onset with the SGA. Otherwise, clinicians should consider intermittent use or lower doses when possible in these patient subtypes to minimize the risk of TD and while weighing the consequences and the need of increased monitoring for psychiatric recurrence.

## Conclusion

SGAs are powerful pharmacologic tools used to treat a wide array of psychiatric conditions. Use is not just limited to psychosis but may also extend to mania, depression, anxiety and even autism spectrum disorders. To treat these conditions, most SGAs are dosed daily and used chronically. Evidence to date suggests that the risk of TD is probably lower with SGAs as compared to FGAs, but TD still will occur with SGAs, as seen in the above cases. Prospective, large-scale studies involving antipsychoticnaïve patients exposed to SGAs only would be needed to confirm the true risk and incidence of SGA-induced TD. Regardless, TD will continue to be a side-effect that requires vigilant monitoring.

## Abbreviations

1A2, 2D6/CYP2D6: Cytochrome p450 enzymes
5HT-1A, 5HT-2A: serotonin 1A receptor, serotonin 2A receptor
AAN: American Academy of Neurology
AIMS: abnormal involuntary movement scale
BCAA: branched-chain amino acids
COMT: catechol-O-methyltransferase
CYP450: cytochrome P450
D2, D3: dopamine 2 receptor, dopamine 3 receptor
DAT: dopamine transporter
DRD3 gene: dopamine 3 receptor gene
EGb-761: a ginkgo biloba extract
ECT: electroconvulsive therapy
EPS: extrapyramidal symptoms
FGA: first-generation antipsychotics
GABA: gamma-aminobutyric acid
HTR2A: serotonin receptor gene
MDD: major depressive disorder
MSN: medium spiny neuron
NMDA: N-methyl-D-aspartate
OCD: obsessive-compulsive disorder
OR: odds ratio
RCT: randomised controlled trial
SGA: second-generation antipsychotics
SPECT: single-photon emission computerised tomography
TD: tardive dyskinesia
THIP: tetrahydroisoxazolopyridinol.
